# Diazoxide and moderate‐intensity exercise improve skeletal muscle function by decreasing oxidants and enhancing antioxidant defenses in hypertensive male rats

**DOI:** 10.14814/phy2.16026

**Published:** 2024-04-23

**Authors:** Estefanía Bravo Sánchez, César J. Nolasco Ruíz, Mariana Gómez‐Barroso, Christian Cortés Rojo, Alain R. Rodríguez Orozco, Alfredo Saavedra Molina, Salvador Manzo Ávalos, Rocío Montoya Pérez

**Affiliations:** ^1^ Instituto de Investigaciones Químico‐Biológicas Universidad Michoacana de San Nicolás de Hidalgo Morelia Mexico; ^2^ Facultad de Ciencias Médicas y Biológicas “Dr. Ignacio Chávez” Universidad Michoacana de San Nicolás de Hidalgo Morelia Mexico

**Keywords:** antioxidant, diazoxide, exercise, hypertension, skeletal muscle

## Abstract

High sodium intake is decisive in the incidence increase and prevalence of hypertension, which has an impact on skeletal muscle functionality. Diazoxide is an antihypertensive agent that inhibits insulin secretion and is an opener of K_ATP_ channels (adosine triphosphate sensitive potasium channels). For this reason, it is hypothesized that moderate‐intensity exercise and diazoxide improve skeletal muscle function by reducing the oxidants in hypertensive rats. Male Wistar rats were assigned into eight groups: control (CTRL), diazoxide (DZX), exercise (EX), exercise + diazoxide (EX + DZX), hypertension (HTN), hypertension + diazoxide (HTN + DZX), hypertension + exercise (HTN + EX), and hypertension + exercise + diazoxide (HTN + EX + DZX). To induce hypertension, the rats received 8% NaCl dissolved in water orally for 30 days; in the following 8 weeks, 4% NaCl was supplied to maintain the pathology. The treatment with physical exercise of moderate intensity lasted 8 weeks. The administration dose of diazoxide was 35 mg/kg intraperitoneally for 14 days. Tension recording was performed on the extensor digitorum longus and the soleus muscle. Muscle homogenates were used to measure oxidants using fluorescent probe and the activity of antioxidant systems. Diazoxide and moderate‐intensity exercise reduced oxidants and increased antioxidant defenses.

## 1 INTRODUCTION

Hypertension (HTN) is a major public health issue due to its high prevalence and the significant increase in cardiovascular morbidity and mortality that it entails (Navas‐Santos et al., 2016). The causes of HTN are multiple and involve various risk factor, including alterations in the autonomic system, renal control of vascular nerve resistance and circulating volume, and direct regulation of vascular tone (Zehnder, 2010). The excessive dietary intake of sodium and reduced intake of potassium that characterizes the Western diet, together with obesity and a sedentary lifestyle, favors a progressive increase in the incidence and prevalence of HTN (Zehnder, 2010). On the other hand, excessive sodium enhances endothelial damage due to an intracellular substitution of potassium for sodium, altering endothelial function. Sodium increases in the cerebrospinal fluid trigger sympathetic hyperstimulation and activation of the renin‐angiotensin‐aldosterone system (RAAS). All these factors impair the function of the vascular endothelium, inducing an increase in peripheral vascular resistance and, therefore, HTN (Ekiz et al., 2021; Zehnder, 2010). The increase in blood volume and water retention elicited by overstimulation of the RAAS by sodium triggers a series of responses including low GLUT4 expression, increased protein degradation, apoptosis, and vasoconstriction. In the skeletal muscle, this impaired its functionality and the percentage of muscle mass. In this way, the loss of muscle mass can enhance inflammatory and oxidative pathways (Buckley et al., 2020; Ekiz et al., 2021; Zehnder, 2010).

In skeletal muscle, it is suggested that ATP‐sensitive potassium channels (K_ATP_) are activated during fatigue and metabolic stress. For this reason, their function has acquired great biomedical importance for some diseases (Sánchez‐Duarte et al., 2020). Diazoxide (7‐chloro‐3‐methyl‐4H,1,2,4‐benzothiadiazine 1,1‐dioxide) is a benzothiadiazine derivative and an agonist that increases the opening time of mitoK_ATP_ channels, since it has affinity for the sulphonylurea receptor subunit of the channel (Constant‐Urban et al., 2013). Furthermore, diazoxide is a vasodilator and an inhibitor of insulin secretion. (Alemzadeh et al., 2008) Diazoxide confers cardioprotection in isolated rabbit myocytes (Liu et al., 1998). Activation of K_ATP_ channels improves muscle contraction and promotes fatigue resistance, reducing the levels of reactive oxygen species (ROS) and increasing antioxidant levels. Diazoxide has significant effects on improving muscle contraction force and improving fatigue resistance time (Alemzadeh et al., 2008; Sánchez‐Duarte et al., 2020).

On the other hand, exercise promotes various metabolic and functional adaptations in skeletal muscle (Lambertucci et al., 2006). Exercise attenuates arterial remodeling in spontaneously hypertensive rats (Gu et al., 2013), increases the capillary‐to‐fiber ratio in humans (Gliemann et al., 2015), and improves the balance between pro‐oxidants and antioxidants (Higashi & Yoshizumi, 2004). Exercise training improves endothelial function and conditions for oxygen diffusion in skeletal muscle of hypertensive subjects, and these adaptations are important for better matching of blood flow and oxidative metabolism in exercising muscle (Nyberg et al., 2015). On this basis, we hypothesize that moderate‐intensity exercise in combination with diazoxide improves skeletal muscle function by reducing the levels of oxidants and increasing both noncatalytic (i.e., glutathione) and catalytic antioxidants (i.e., catalase) in hypertensive rats.

## 2 MATERIALS AND METHODS

### 2.1 Experimental groups

**FIGURE 1:**
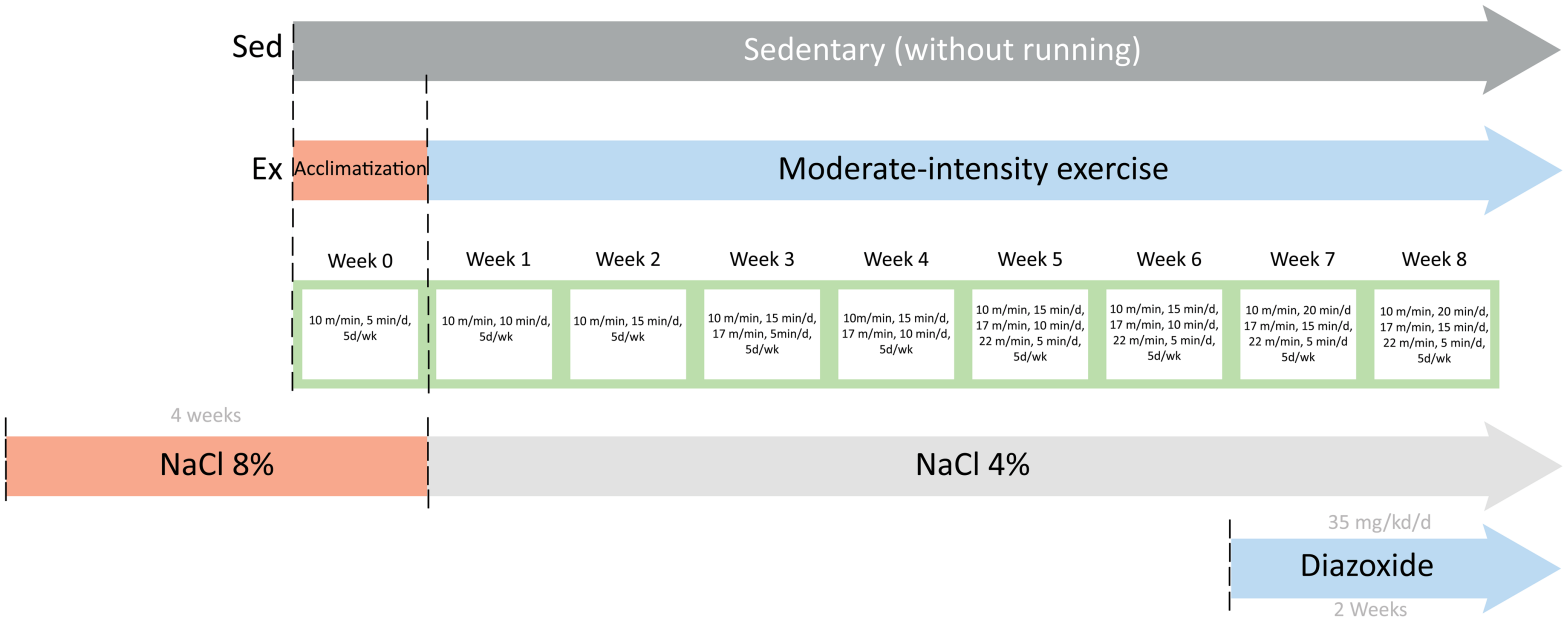
Experimental design.

Male Wistar rats of 250 h were housed and maintained at 24°C with 12‐h day/night cycles, fed a standard rodent diet (Rodent Diet 5001, SA de CV, México), and given water ad libitum. All animal procedures were conducted following the technical specifications for the production, use, care, and handling of laboratory animals (NOM‐062‐ZOO‐1999, Ministry of Agriculture, México City, México) and were approved by the Institutional Committee for the Use and Care of Animals of the Instituto de Investigaciones Químico‐Biológicas of the Universidad Michoacana de San Nicolás de Hidalgo. The animals were randomly assigned to experimental groups, with *n* = 6 per group; were divided into eight groups: control (CTRL), hypertensive (HTN), diazoxide (DZX), exercise (EX), exercise + diazoxide (EX+DZX), hypertensive + exercise (HTN + EX), hypertensive + diazoxide (HTN + DZX) and hypertensive + exercise + diazoxide (HTN + EX + DZX). Some of the rats did not survive before subsequent measurements of blood pressure and metabolic biomarkers; thus, *n* was reduced to 5. The dose of diazoxide (D9035, Sigma‐Aldrich, St. Louis MO, USA) was 35 mg/kg by intraperitoneal injection daily for 14 days at the end of the protocols. For hypertensive groups, rats received 8% NaCl dissolved in water orally for 30 days to induce HTN. In the following 8 weeks, 4% NaCl (JT Baker, Phillipsburg, NJ, USA) was supplied to maintain the pathology (Jakovljevic et al., 2019). The exercised groups were subjected to an exercise protocol for 8 weeks, described in Figure 1 (Gómez‐Barroso et al., 2020). The training was always performed at the beginning of the active/dark cycle (18–20 h), as the animals showed better exercise tolerance during the dark cycle. Untrained animals were not exposed to exercise of any kind and the animals that were trained finished each training section.

### 2.2 Blood pressure measurement

Blood pressure was measured in conscious animals by a noninvasive method (tail‐cuff) using a CODA monitor single channel noninvasive blood pressure system (Kent Scientific, Torrington, CT, USA). The rats were placed on a holder 5 min before the measurements, and three recordings of systolic and diastolic pressures were made for each animal every 2 weeks throughout the treatment. All procedures were performed according to the manufacturer's instructions.

### 2.3 Metabolic biomarkers

Weight and basal glucose were monitored in the groups after 12 h fasting using a scale and an AccuCheck® Perfoma glucometer, respectively, once a week at 9:00 am, during the weeks of the experimental protocol.

### 2.4 Insulin tolerance test

At the end of the experimental protocol, insulin tolerance test (ITT) was evaluated in all groups. Rats were fasted for 15 h, and basal blood glucose concentration was determined with blood from the tail tip using a glucometer (Accu‐Chek® Performa, Roche, Indianapolis, IN, USA). Blood glucose levels were determined at 0, 30, 60, 90, and 120 min after an intraperitoneal insulin injection (0.75 U insulin/kg body weight) (398 M94, SSA IV, Laboratorios Pisa, S.A. de CV, Guadalajara Jal., México). The glucose disappearance rate (KITT), derived from the ITT, was calculated by dividing 0.693 by the plasma glucose half‐time (t1/2) × 100 as described previously (Inchiostro, 2005; Monzillo & Hamdy, 2003). The plasma glucose t1/2 was calculated from the slope of least square analysis of the glucose concentrations after i.p. insulin injection during the linear phase of decline. This test indicates the action of insulin in periphery tissues, showing their sensitivity to the hormone (Vinué & González‐Navarro, 2015).

### 2.5 Muscle extraction and tissue preparation

The day after carrying out the ITT, the rats were sacrificed by decapitation. The Extensor Digitorium Longus (EDL) and Soleus muscles were dissected and obtained from both hindlimbs. The muscles of one of the limbs were placed and fixed with entomological pins in a Petri dish lined with a transparent resin bottom (Sylgard); this was previously filled with Krebs–Ringer saline solution (118 mM NaCl, 4.75 mM KCl, 1.18 mM MgSO_4_, 24.8 mM NaHCO_3_, 1.18 mM KH_2_PO_4_; pH 7.4) plus an addition of 10 mM glucose and constant carbogen gas (95% O_2_ and 5% CO_2_) was also supplied and cleaning was performed with the aid of a stereoscopic microscope of the mounted muscles to remove excess connective tissue and fatty tissue adhered to the muscle when performing the dissection, to be able to record tension finally. On the other hand, the other hindlimb muscles were stored in a deep‐frozen Krebs–Ringer saline solution (−80°C). After preserving the tissue, a homogenate was made for the corresponding biochemical tests.

### 2.6 Isometric tension measurements

Soleus and EDL muscles were kept submerged and perfused with Krebs–Ringer solution (118 mM NaCl, 4.75 mM KCl, 1.18 mM MgSO_4_, 24.8 mM NaHCO_3_, 1.18 mM KH_2_PO_4_, 10 mM glucose, and CaCl_2_ 2.54 mM) and carbogen gas (95% O_2_ and 5% CO_2_). The muscle was cleaned and mounted as indicated in the methodology (Gómez‐Barroso et al., 2020). The muscle was stretched to 1.3 times its resting length and left to perfuse in the physiological solution for 10 min before recording the isometric tension; the experiment was performed at a temperature of 25 ± 1°C. For maximum and total isometric twitch tension measurements, the recording chamber was connected to an optical force transducer, which, through an amplifier and an analog–digital interface (World Precision Instruments, Sarasota, FL, USA), allowed acquiring the tension generated by the muscle in a computer, using MDAC (channel data acquisition system) software (World Precision Instruments, Sarasota, FL, USA). Muscles were activated by supramaximal stimulation via platinum electrodes placed in a parallel direction to the muscle's longitudinal axis. Two platinum electrodes were placed inside the recording chamber, which was connected to a stimulus isolation unit and an electric stimulator (Grass). The muscle was repeatedly stimulated using an electric current to produce multiple isometric contractions over a period of time by applying 100 V pulses, 300 ms in duration, at the frequency of 45 Hz for soleus muscle and 50 Hz for EDL muscle; the electrical stimulation was generated until the muscle was fatigued (~70% reduction in the initial strength).

### 2.7 Measurement of reactive oxygen species

Oxidants levels were determined using the cell‐permeable fluorescent probe 2′,7′‐dichlorodihydrofluorescein diacetate (H_2_DCFDA) (D6883, Sigma‐Aldrich, St. Louis, MO, USA). In this study, 0.5 mg of protein was placed in 2 mL of buffer containing 100 mM KCl, 10 mM HEPES, 3 mM KH_2_PO_4_, and 3 mM MgCl_2_ (pH 7.4) and incubated with 12.5 μM of H_2_DCFDA for 15 min in an ice‐bath under constant shaking and the fluorescence was recorded at 0 and 60 min at 491 nm (ext) and 518 nm (em), in a spectrofluorophotometer (Shimadzu RF‐5301PC, Kyoto, Japan) (expressed as units/mg of protein).

### 2.8 Determination of glutathione status

Total glutathione, GSSG (oxidized glutathione), and GSH (reduced glutathione) were determined using an enzymatic recycling method, according to (Huerta‐Cervantes et al., 2020). In brief, 0.5 mg protein of muscle homogenate was resuspended in a blend containing 0.1% Triton‐X, 0.6% sulfosalicylic acid, and 5 mM Na_2_EDTA in 50 mM potassium phosphate buffer. The samples were sonicated in 3 cycles (sonication/ice) in a Branson sonifier 450 with a tapered microtip (20 W output/constant duty cycle); next, the samples were subject to two repeated freeze/thaw cycles and centrifugated for 10 min at 7200 rpm. Later, 100 μL of the supernatant was placed in a mixture containing 5 mM Na_2_EDTA, 0.1 mM 5,5′‐dithiobis‐2‐nitrobenzoic acid (D8130, Sigma‐Aldrich, St. Louis, MO, USA) and 100 mU/mL glutathione reductase in 50 mM potassium phosphate buffer and incubated for 1 min at room temperature. Then, the reaction was started by the addition of 50 μM β‐NADPH (N6506, Sigma‐Aldrich, St. Louis, MO, USA). The change in absorbance was registered at 412 nm at 30°C in a UV/vis spectrophotometer (Shimadzu UV‐2550, Kyoto, Japan), and changes in absorbance were recorded for 5 min. GSSG from the different samples was determined after incubation with 0.2% 4‐vinyl pyridine (V3204, Sigma‐Aldrich, St. Louis, MO, USA) at room temperature for 1 h. GSH from the different samples was calculated by subtracting GSSG from the total glutathione, and the GSH/GSSG ratio was calculated by dividing the GSH levels by the GSSG levels.

### 2.9 Determination of catalase activity

Catalase activity was assayed by measuring the conversion of H_2_O_2_ into O_2_ using a Clark‐type oxygen electrode connected to a biological oxygen monitor YSI 5300A (Yellows Springs, OH, USA), according to (Foster & Coetzee, 2016), with slight modification. Briefly, 0.5 mg of protein of muscle homogenate was resuspended in a 0.1 M potassium phosphate buffer (pH 7.6) at 25°C, and the trace was monitored for 1 min. Later, fresh 6 mM of H_2_O_2_ was added to the chamber, and the conversion of H_2_O_2_ to oxygen was recorded for 2 min. Finally, 1.0 mM of sodium azide (S2002, Sigma‐Aldrich, St. Louis, MO, USA) was added to the chamber. Catalase activity was calculated using bovine catalase as standard (expressed as AU/mg of protein).

### 2.10 Statistical analysis

The study was designed to detect differences in normotensives and hypertensive groups. The assumptions of normality and equal variance were tested. The primary outcomes were compared using factorial analysis of variance (ANOVA) (Tables S1–S25) to compare the means of three independent categorical variables (hypertension × exercise × drug). Tukey multiple comparisons were completed when interactions were detected. Statistical significance was set at *p* < 0.05. Data are expressed as mean and standard deviation with individual data points displayed when possible. All statistical analyses were performed using the software STATISTICA v 8.0® (Statsoft) and Prism v.8.0.1 software (GraphPad Inc., La Jolla, CA, USA).

## 3 RESULTS

### 3.1 Effects of diazoxide and exercise on blood pressure, weight, and blood glucose levels

**FIGURE 2:**
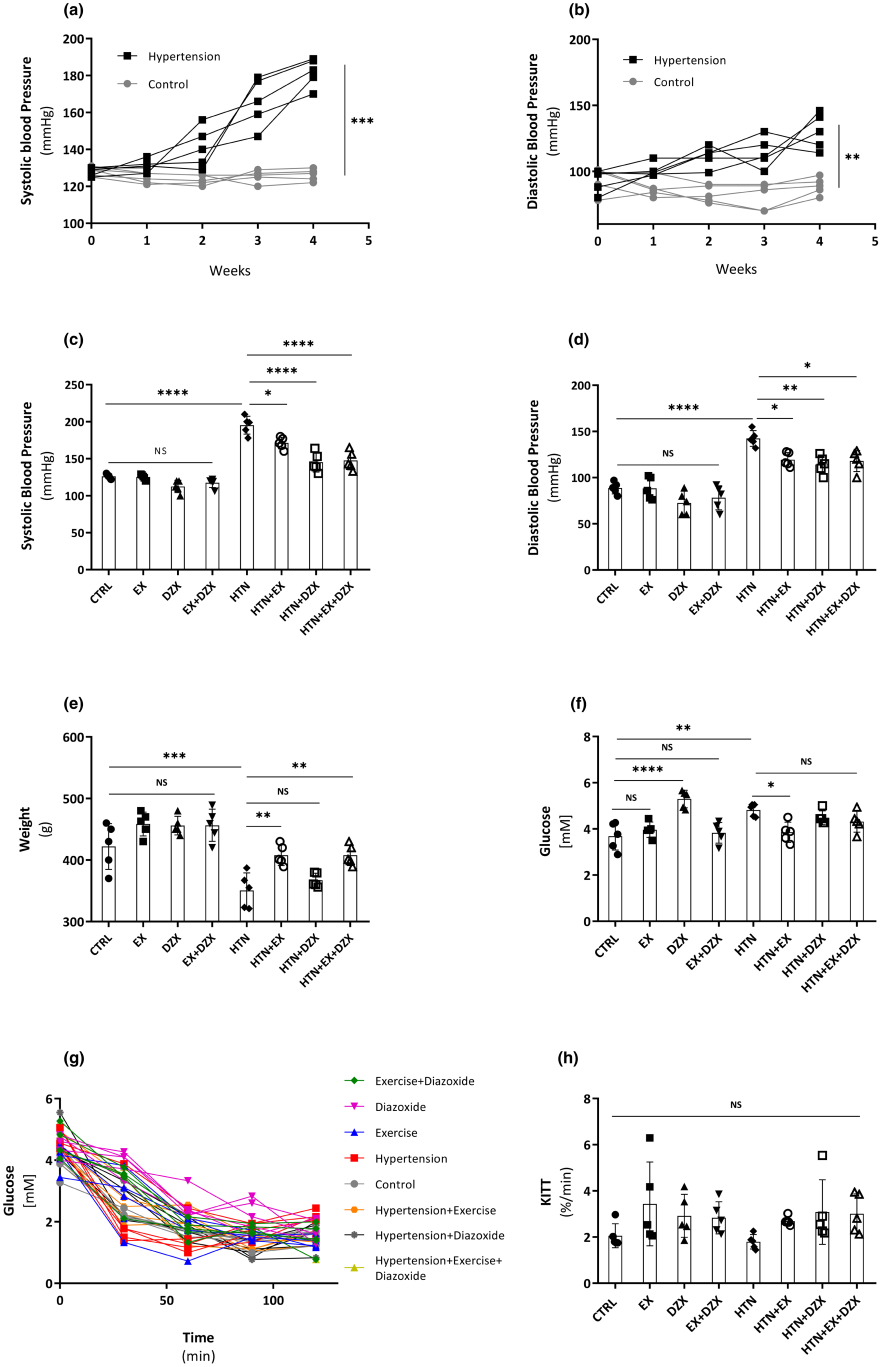
The effect of diazoxide and exercise on metabolic biomarkers. This figure shows the effect of NaCl on systolic blood pressure (a) levels (mmHg); and diastolic blood pressure (b) levels (mmHg); the effect of exercise and diazoxide on systolic blood pressure (c) levels (mmHg); diastolic (d) blood pressure levels (mmHg); body weight (e) (g); fasting glucose levels (f) [mM]; insulin tolerance test (ITT) glucose levels (g) [Mm]; glucose rate disappearance (KITT) during the ITT (h) (%/min). CTRL, control; EX, exercise; DZX, diazoxide; EX+DZX, exercise + diazoxide; HTN, hypertension; HTN + EX: hypertension + exercise; HTN + DZX, hypertension + diazoxide; HTN + EX+DZX, hypertension + exercise + diazoxide. The data are expressed as mean ± standard deviation (SD); *n* = 6. Effects were evaluated using factorial ANOVA. When interactions were detected, Tukey post hoc paired comparisons were completed and reported such that *p < 0.05; **p < 0.01; ***p < 0.001; ****p < 0.0001.

The treatment with 8% NaCl increased systolic (Figure 2a) and diastolic (Figure 2b) blood pressure in the fourth week (*p* ≤ 0.0002 and *p* ≤ 0.003, respectively). The systolic blood pressure at the end of the treatments (Figure 2c) shows that NaCl‐treated rats remained hypertensive (*p* ≤ 0.0001). In contrast, the hypertensive + exercise group exhibited a decrease in systolic pressure in comparison to the hypertensive group (*p* < 0.01). In turn, the hypertensive + diazoxide (*p* ≤ 0.0001) and hypertensive + exercise + diazoxide (*p* < 0.0001) groups presented lower systolic blood pressure levels than the remaining experimental groups. Regarding the changes in diastolic blood pressure levels, Figure 2d shows that the NaCl‐treated rats exhibited significantly elevated pressure values (*p* ≤ 0.0001). Conversely, the hypertensive + exercise (*p* ≤ 0.03), hypertensive + diazoxide (*p* ≤ 0.004), and hypertensive + exercise + diazoxide (*p* ≤ 0.01) groups displayed attenuated values of diastolic blood pressure in comparison to the hypertensive group.

The body weight at the end of treatment (Figure 2e) was lower in the hypertensive group than in the control group. In contrast, the body weight was restored in the hypertensive + exercise (*p* ≤ 0.008) and hypertensive + exercise + diazoxide (*p* ≤ 0.006) groups, although this effect was not observed in the hypertensive + diazoxide group.

Fasting blood glucose levels are depicted in Figure 2f. The hypertensive (*p* ≤ 0.005) and diazoxide (*p* ≤ 0.0001) groups had higher glucose levels than the control group. Moreover, it was observed that glucose levels in the hypertensive + diazoxide and hypertensive + exercise + diazoxide groups were not different in comparison to the hypertensive group. In contrast, the hypertensive + exercise group presented (*p* ≤ 0.02) a decrease in fasting glucose levels compared to the hypertensive group. Changes in insulin sensitivity were evaluated with an ITT (Figure 2g). From these data, the glucose disappearance rate (KITT) was calculated (Figure 2h). No statistical differences were observed between all the experimental groups.

### 3.2 Effects of diazoxide and exercise on muscle contraction force

**FIGURE 3:**
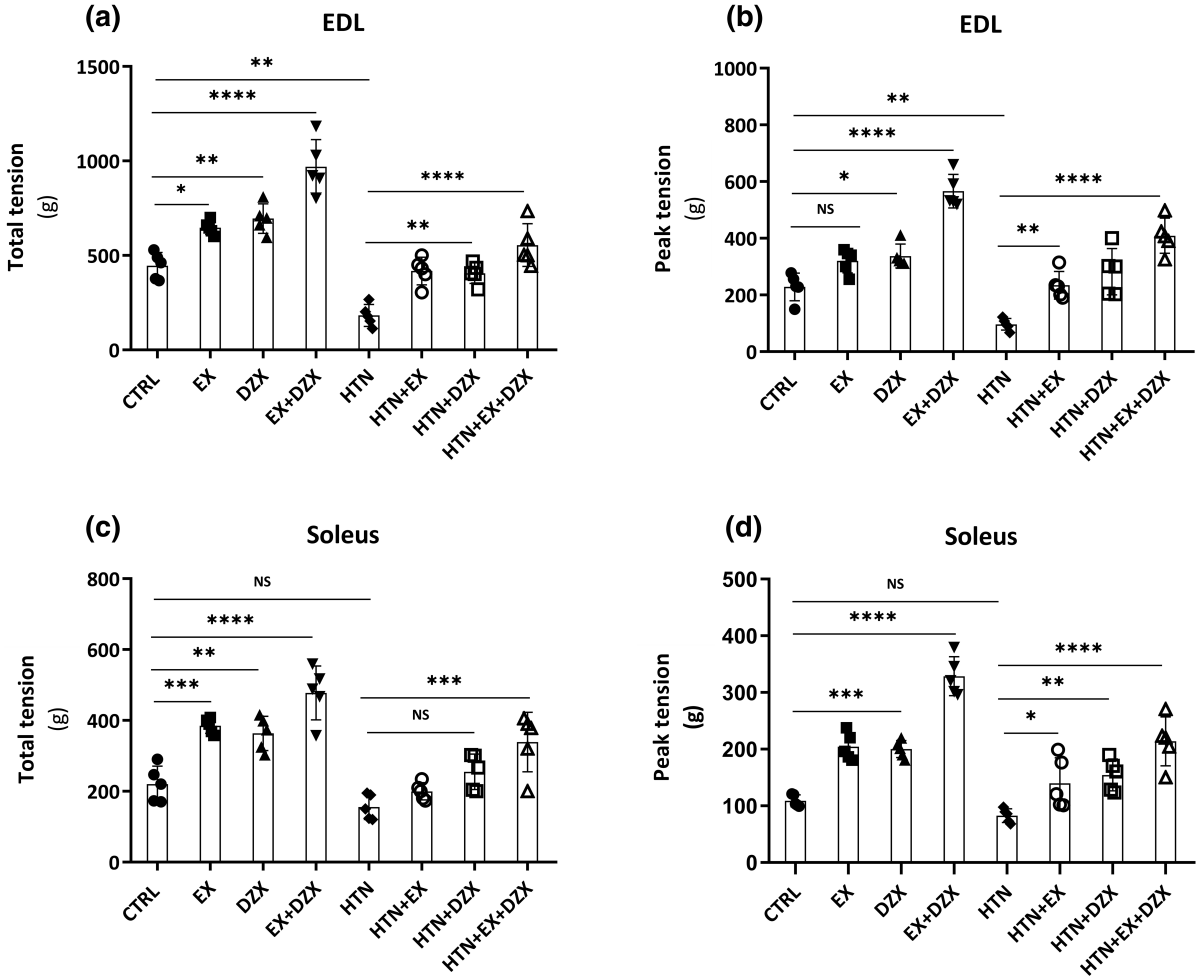
Effect of diazoxide and exercise on total and maximum tension. This figure shows the total tension in the EDL muscle (a) (in grams), the maximum tension in the EDL muscle (b) (in grams), the total tension in the soleus muscle (c) (in grams), and the maximum tension in the soleus muscle (d) (in grams). The data are expressed as mean ± standard deviation (SD); *n* = 5. Effects were evaluated using factorial ANOVA. CTRL, control; DZX, diazoxide; EX + DZX, diazoxide + exercise; EX, exercise; HTN + DZX, hypertension + diazoxide; HTN + EX + DZX, hypertension + exercise + diazoxide; HTN + EX, hypertension + exercise; HTN, hypertension. When interactions were detected, Tukey post hoc paired comparisons were completed and reported such that *p < 0.05; **p < 0.01; ***p < 0.001; ****p < 0.0001.

Figure 3a shows the total tension of the EDL muscle. Total tension was lower in the hypertensive group in contrast to the control group, reflecting that HTN impairs the function of the EDL muscle. The hypertensive + exercise + diazoxide group (*p* ≤ 0.0001) displayed improved muscle contraction force compared to the hypertensive group, while exercise or diazoxide separately increased total tension, although not to the same extent as the combination of these two treatments. Figure 3b shows that changes in maximum tension in the EDL muscle follow a similar behavior to that observed in total tension in the same type of muscle.

Figure 3c shows the total tension of the soleus muscle. The total tension was not different between the control and hypertensive groups. Nevertheless, it can be noted that muscle contraction force in the hypertensive + exercise + diazoxide group (*p* = 0.0001) was higher in comparison to the hypertensive group. The maximum tension in the soleus muscle (Figure 3d) did not show significant differences in the hypertensive group in contrast to the control group; however, with the exercise + diazoxide interventions, the force of muscle contraction in the maximum tension increased in the soleus muscle.

**FIGURE 4:**
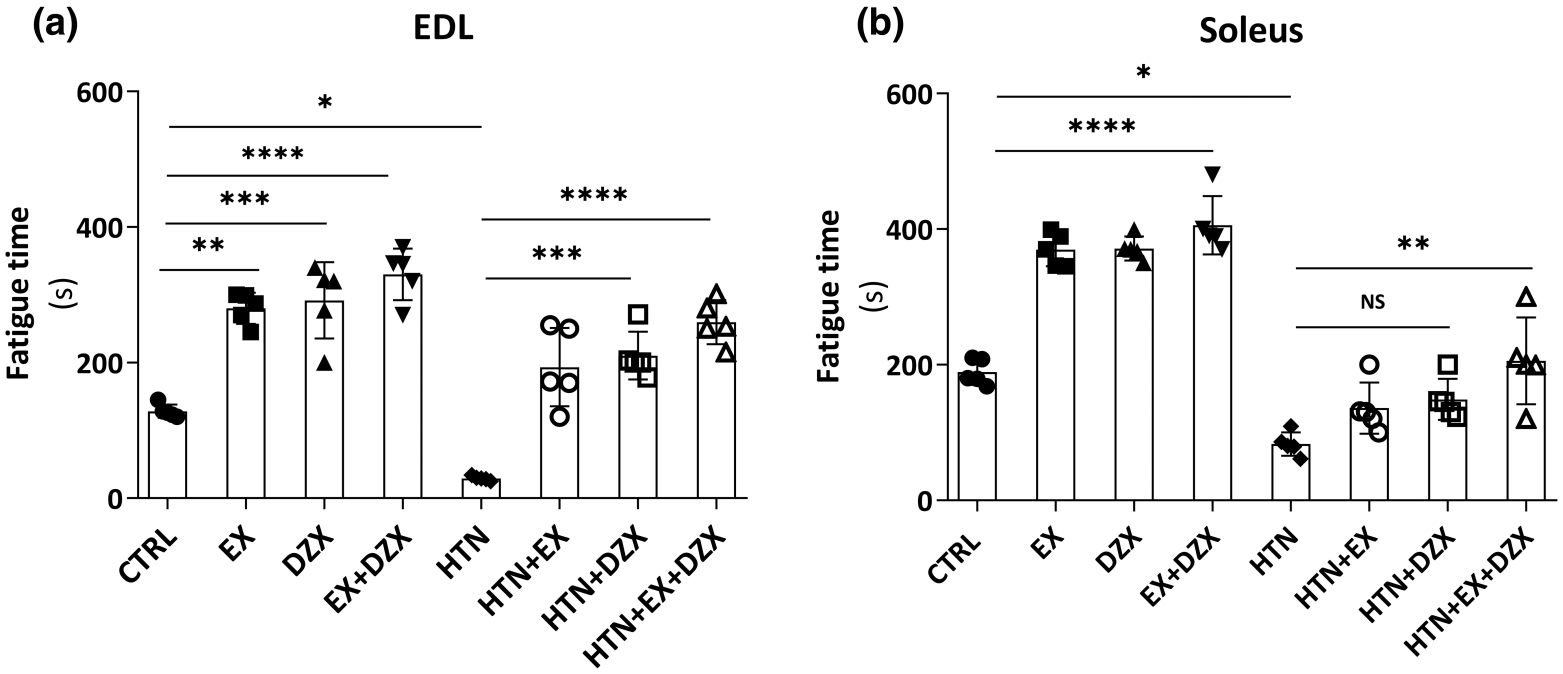
Effect of diazoxide and exercise on fatigue resistance time. This figure shows the fatigue resistance time of the EDL muscle (a) (in seconds), the fatigue resistance time of the soleus muscle (b) (in seconds). The data are expressed as mean ± standard deviation (SD); *n* = 5. Effects were evaluated using factorial ANOVA. CTRL, control; DZX, diazoxide; EX + DZX, diazoxide + exercise; EX, exercise; HTN + DZX, hypertension + diazoxide; HTN + EX + DZX, hypertension + exercise + diazoxide; HTN + EX, hypertension + exercise; HTN, hypertension. When interactions were detected, Tukey post hoc paired comparisons were completed and reported such that *p < 0.05; **p < 0.01; ***p < 0.001; ****p < 0.0001.

Figure 4a displays the fatigue resistance time in the EDL muscle. The time of resistance to fatigue decreased in the hypertensive group (*p* ≤ 0.05) when compared to the control group. On the other hand, fatigue resistance time improved in HTN + diazoxide and hypertension + exercise groups in contrast to the hypertensive group. However, it should be noted that the hypertensive + exercise + diazoxide group (*p* ≤ 0.0001) had an even greater increase in fatigue resistance time.

The fatigue resistance time in the soleus muscle was lower in the hypertensive group (*p* ≤ 0.01) compared to the control group (Figure 4b). On the other hand, the HTN + exercise + diazoxide group (*p* ≤ 0.005) presented an increase in the time of resistance to fatigue compared to the hypertensive group.

### 3.3 Effects of diazoxide and exercise on oxidants levels and antioxidants in muscle of hypertensive rats

**FIGURE 5:**
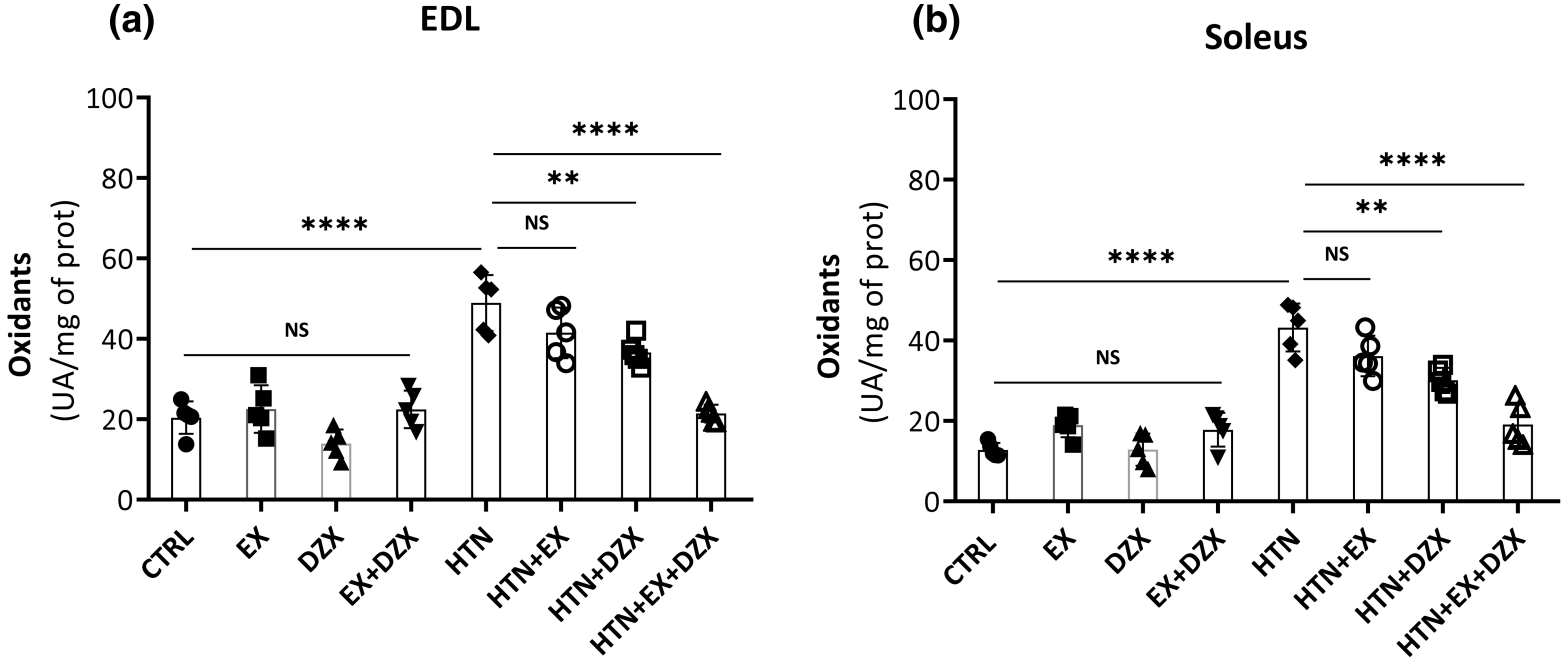
Effect of diazoxide and exercise on oxidants levels in skeletal muscle. This figure shows the levels of oxidants (U of activity/mg of prot) in the EDL muscle (a) and the soleus muscle (b) (U of activity/mg of prot). CTRL, control; EX, exercise; DZX, diazoxide; EX + DZX, exercise + diazoxide; HTN, hypertension; HTN + EX, hypertension + exercise; HTN + DZX, hypertension + diazoxide; HTN + EX + DZX, hypertension + exercise + diazoxide. The data are expressed as mean ± standard deviation (SD); *n* = 5. Effects were evaluated using factorial ANOVA. When interactions were detected, Tukey post hoc paired comparisons were completed and reported such that *p < 0.05; **p < 0.01; ***p < 0.001; ****p < 0.0001.

The levels of oxidants in the EDL muscle are shown in Figure 5a. Oxidants production increased 139.8% in the muscle of the hypertensive group in comparison to the control group. No statistically significant differences are observed between the hypertensive + exercise group and the hypertensive group. In contrast, there was a decrease in oxidant levels in the HTN + diazoxide group (*p* ≤ 0.005) when compared to the hypertensive group. This decrease in oxidants levels was more accentuated in the hypertensive + exercise + diazoxide group (*p* ≤ 0.0001) in comparison to the hypertensive group. Figure 5b shows the levels of oxidants in the soleus muscle of hypertensive rats. There was an increase in oxidants production in the hypertensive group (*p* ≤ 0.0001) compared to the control group. On the contrary, the diazoxide + exercise + hypertensive group presented a statistically significant decrease in oxidants production (*p* ≤ 0.0001) compared to the hypertensive group.

**FIGURE 6:**
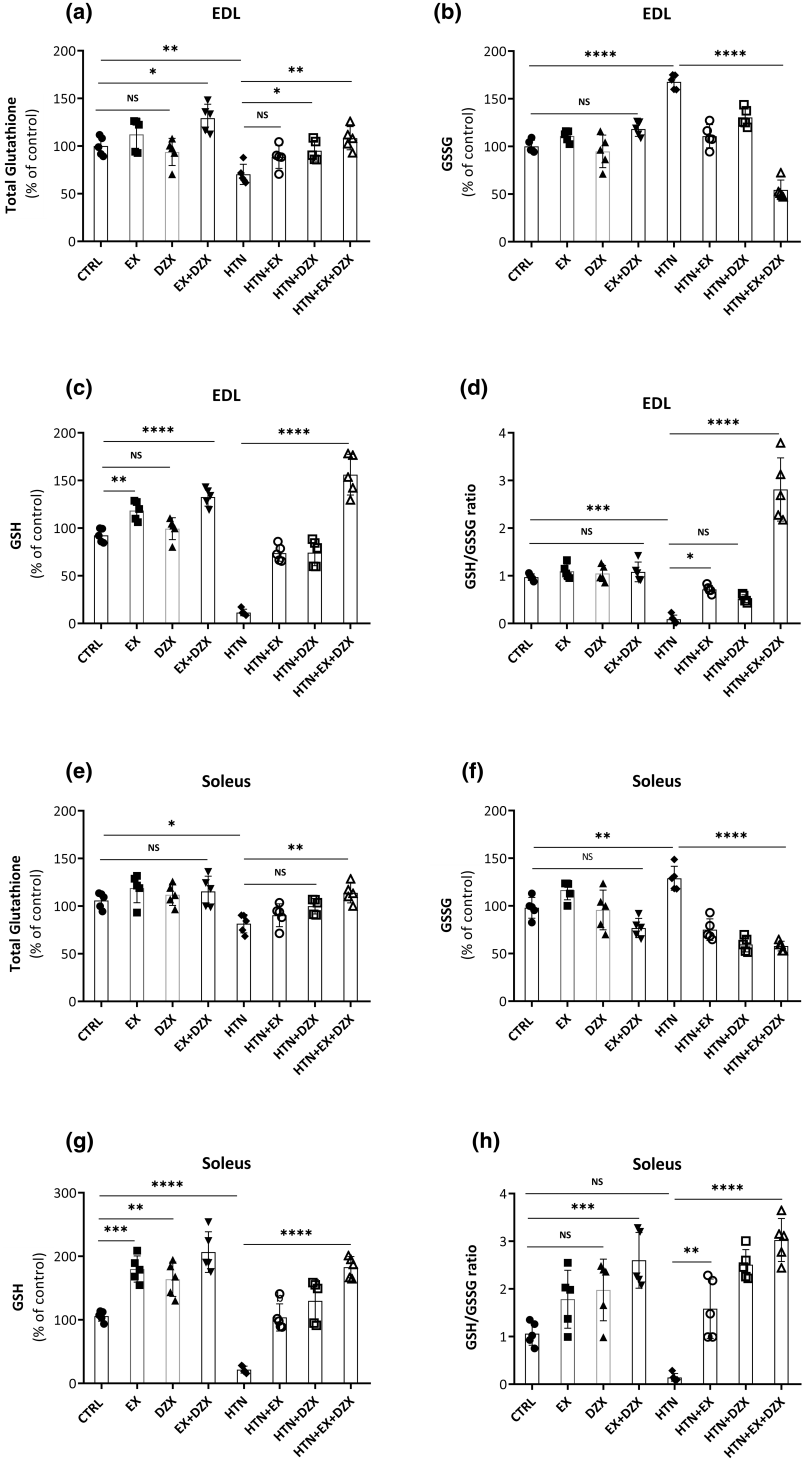
The effect of diazoxide and exercise on glutathione status in skeletal muscle. This figure shows the levels of total glutathione in the EDL muscle (a), oxidized glutathione in the EDL muscle (b), reduced glutathione in the EDL muscle (c), GSH/GSSG ratio in the EDL muscle (d), glutathione total in the soleus muscle (e), oxidized glutathione in the soleus muscle (f), reduced glutathione in soleus muscle (g), and GSH/GSSG ratio in the soleus muscle (h). CTRL, control; EX, exercise; DZX, diazoxide; EX + DZX, exercise + diazoxide; HTN, hypertension; HTN + EX: hypertension + exercise; HTN + DZX, hypertension + diazoxide; HTN + EX + DZX, hypertension + exercise + diazoxide. The data are expressed as mean ± standard deviation (SD); *n* = 5. Effects were evaluated using factorial ANOVA. When interactions were detected, Tukey post hoc paired comparisons were completed and reported such that *p < 0.05; **p < 0.01; ***p < 0.001; ****p < 0.0001.

Regarding the antioxidant systems, it was analyzed the redox state of glutathione in both the EDL and soleus muscle. The levels of total glutathione in the EDL muscle (Figure 6a) were lower in the hypertensive group (*p* ≤ 0.009) in contrast to the control group. Oxidized glutathione (GSSG) (Figure 6b) was higher in the EDL muscle of the hypertensive group (*p* ≤ 0.0001) in comparison to the control group. On the other hand, the GSSG levels in the hypertension + exercise + diazoxide group (*p* ≤ 0.0001) were significantly lower compared with the hypertensive group. Consistently, the levels of reduced glutathione (GSH) in the EDL muscle (Figure 6c), were higher in the hypertension + exercise and hypertension + diazoxide groups in comparison to the hypertensive group (*p* ≤ 0.0001). These differences in GSH and GSSG levels led to a drastic decrease in the GSH/GSSG ratio in the EDL muscle of the hypertensive group in comparison to the control group (Figure 6d), which was counteracted in the hypertensive + exercise, hypertensive + diazoxide, and hypertensive + exercise + diazoxide groups (*p* ≤ 0.0001), being of greater magnitude in the latter group.

Total glutathione levels in the soleus muscle (Figure 6e) of the hypertensive group were statistically significant lower compared to the control group (*p* ≤ 0.03). This was counteracted only in the hypertension + exercise + diazoxide group. Oxidized GSSG content (Figure 6f) increased in the hypertensive group in comparison to the control group. In contrast, in comparison to the control group, there was a decrease in hypertension + exercise, hypertension + diazoxide, and hypertension + exercise + diazoxide groups (*p* ≤ 0.0001). Figure 6g shows the GSH values in the soleus muscle. GSH content in the hypertensive group was lower than in the control group (*p* ≤ 0.0001). The hypertension + exercise + diazoxide group had higher GSH content (*p* ≤ 0.0001) than the hypertensive group. In turn, as in the EDL muscle, the GSH/GSSG ratio in the soleus muscle was ostensibly lower in the hypertensive group than in the control group (Figure 6h), being this effect fully counteracted in the hypertension + exercise + diazoxide group (*p* ≤ 0.0001).

**FIGURE 7:**
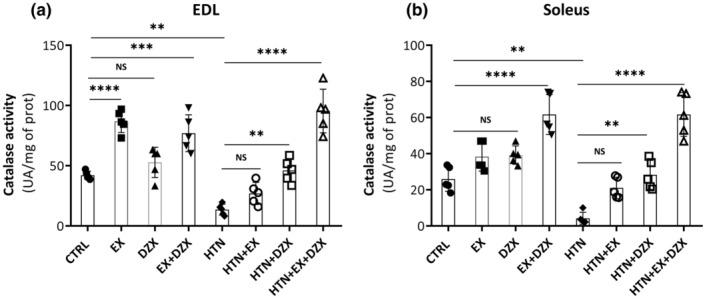
The effect of diazoxide and exercise on catalase activity in skeletal muscle. This figure shows the activity of the catalase enzyme (U of activity/mg of prot) in the EDL muscle (a) and the soleus muscle (b) (U of activity/mg of prot). CTRL, control; EX, exercise; DZX, diazoxide; EX + DZX, exercise + diazoxide; HTN, hypertension; HTN + EX: hypertension + exercise; HTN + DZX, hypertension + diazoxide; HTN + EX+DZX, hypertension + exercise + diazoxide. The data are expressed as mean ± standard deviation (SD); *n* = 5. Effects were evaluated using factorial ANOVA. When interactions were detected, Tukey post hoc paired comparisons were completed and reported such that *p < 0.05; **p < 0.01; ***p < 0.001; ****p < 0.0001.

Figure 7a shows catalase activity in EDL muscle. Catalase activity decreased ostensibly in the hypertensive group (*p* ≤ 0.008) compared to the control group. Notably, catalase activity was enhanced 606% in the hypertension + exercise + diazoxide group, in contrast to the hypertensive group. These results are corroborated in the soleus muscle (Figure 7b), where a similar behavior in catalase activity was observed in contrast to the EDL muscle, with a notable decrease in catalase activity in the hypertensive group (*p* ≤ 0.005) in contrast to the control group, and an increase in 1359% in catalase activity in the hypertension + exercise + diazoxide group in comparison with the hypertensive group.

## 4 DISCUSSION

In skeletal muscle, moderate‐intensity exercise promotes glucose transport, increases the levels of antioxidants, and decreases ROS levels by inducing the opening of K_ATP_ channels, driving improved muscle function (Jakovljevic et al., 2019; Lambertucci et al., 2006; Sánchez‐Duarte et al., 2020). To our knowledge, this is the first report investigating the probable beneficial effects of moderate‐intensity exercise and the pharmacological opening of K_ATP_ channels in impaired muscle function in sodium‐induced hypertension in the context of muscle redox state.

Eight percent NaCl administration for 4 weeks induced hypertension (Figure 2), which is in agreement with hypertension induction with 8% NaCl in Wistar rats in a previous study (Jakovljevic et al., 2019). It was observed an improvement in blood pressure in hypertensive rats treated with diazoxide plus moderate‐intensity exercise (Figure 2a–d). This effect may be related to enhancement in vascular function by improving the balance between the vasodilator and vasoconstrictor factors, favoring the former. This may be mediated by the opening of endothelial K_ATP_ channels, which mediates the endothelial synthesis of nitric oxide and decreases the production of oxidants (Foster & Coetzee, 2016; Nyberg et al., 2015).

Body weight and glucose were negatively affected in hypertensive rats (Figure 2e–h). This was consistent with a previous report using rats supplemented with 8% NaCl, where, at 16 weeks, observed an increase in food intake and urine volume and a decrease in body weight (Wang et al., 2018). Moreover, it has been reported that body weight decreases in hypertensive rats due to loss of muscle mass (Nemoto & Goyagi, 2021). Weight loss was counteracted by moderate‐intensity exercise (Figure 2e), suggesting that exercise attenuates muscle mass loss in sodium‐induced hypertensive rats. Weight loss in hypertensive animals is known to be accompanied by several changes involving alterations in energy expenditure, lipid metabolism, and hormonal pathways in appetite regulation (Sumithran & Proietto, 2013). Other mechanisms that may be involved in weight recovery in sodium‐induced hypertensive rats may be an increase in appetite, as glycogen depletion, the release of endorphins, and the reduction of blood glucose levels during chronic exercise can increase appetite (Thivel et al., 2019).

Our results showed that sodium‐induced hypertension increases fasting blood glucose levels (Figure 2), which is in agreement with another work showing a pathophysiological relationship between hypertension and insulin resistance (Reaven, 2011). On the other hand, we observed a significant increase in glucose levels with diazoxide in the last 2 weeks of treatment (Figure 2f), which may be related with the inhibitory effect of diazoxide on insulin secretion. Additionally, the ITT was carried out as the data from this assay reflects the action of insulin in peripheral tissues, allowing an estimation of insulin sensitivity (Vinué & González‐Navarro, 2015).

Additional analyses were performed to assess insulin sensitivity, which was determined by measuring the glucose disappearance rate (KITT) derived from the ITT. Experimental evidence in animal models indicates that KITT values are useful in predicting the efficacy of insulin sensitizers (Kozawa et al., 2010; Vardi & Morad, 2001). In our work, no statistically significant difference was shown between all the experimental groups, indicating that insulin sensitivity was not modified by sodium‐induced hypertension diazoxide, either by diazoxide or exercise.

Research on skeletal muscle during hypertension is scarce, and the great diversity of physiological processes affected by this disease remains unknown. Despite there being no differences in tension in the soleus muscle between the hypertensive and control groups (Figure 3), the time of resistance to fatigue was lower in the hypertensive group in comparison to the control group (Figure 4). This observation agrees with a previous report where muscle force was diminished in hypertensive rats (Nemoto & Goyagi, 2021). It has also been determined that hypertension induces the loss of type 1 and type 2 muscle fibers, where a dominant effect was detected on type 2 fibers (Lexell et al., 1988). This may explain why we observed a significant decrease in muscle contraction force in the EDL muscle but not in the soleus muscle. These results corroborated the differences between the soleus muscle and the EDL muscle since the soleus muscle, formed mainly by type I slow‐twitch fibers, develops relatively low tensions and is resistant to fatigue, while the EDL muscle is mainly formed by type II fibers that are fast‐twitch, develop high tensions and depend on anaerobic metabolism, are very susceptible to fatigue (López‐Chicharro & Fernández‐Vaquero, 2010). It should be highlighted that, in our hands, diazoxide and exercise improved both skeletal muscle strength and fatigue resistance time. These results confirm our hypothesis that exercise attenuates muscle fatigue caused by hypertension and it agrees with the data from another study demonstrating that exercise increases the proportion of capillary‐to‐fiber ratio in humans (Gliemann et al., 2015). This adaptation in the capillary ultrastructure improves the conditions of oxygen diffusion and the exchange of metabolites. In addition to these structural changes, in humans, exercise improves endothelial function (Nyberg et al., 2012), improves the balance between prostacyclin and thromboxane levels in skeletal muscle (Hellsten et al., 2012), a better balance between ROS formation and antioxidant systems (Higashi & Yoshizumi, 2004), and lower circulating levels of ET‐1 (Nyberg et al., 2012; Nyberg et al., 2013).

Diazoxide is one of the most used mitoK_ATP_ channel activators (Costa & Garlid, 2008). Activation of K_ATP_ channels improves muscle force production and enhances fatigue resistance time (Moghtadaei et al., 2012). It has been suggested that these effects of diazoxide are mediated by the entry of K^+^ into the mitochondrial matrix, causing the expansion of the mitochondrial volume, increment in mitochondrial respiration, and increased ATP synthesis (Costa & Garlid, 2008).

HTN hypertension courses with endothelial dysfunction, increased levels of proinflammatory cytokines, and oxidative stress (Hernández et al., 2011). We have measured the levels of oxidants in this work with the fluorescent probe DCFH‐DA. This probe was used as a fluorescent probe for ROS measurement despite being oxidized by several types of oxidants. This has raised some concerns about its use as an ROS probe due to its lack of specificity. Nevertheless, DCFH‐DA is still considered as a useful probe for oxidative stress studies in biological systems (Chen et al., 2010). DCFH oxidation is nonspecific and competes with endogenous antioxidants. It can be used as a probe for a type of ROS under certain controlled conditions, but it has also been considered more as a suitable marker for total oxidant production (Chen et al., 2010; Murphy et al., 2022). Thus, this assay may be applied as a qualitative marker of cellular oxidant stress (Chen et al., 2010).

Hypertensive rats show increased oxidant levels (Figure 5), which were counteracted by diazoxide and exercise in both the EDL and soleus muscles. Accordingly, it has been found in spontaneously hypertensive rats an inflammatory process mediated by increased levels of TNF‐α, nitrotyrosine, and iNOS. Oxidative stress was manifested by a decrease in constitutive NOS (nitric oxide synthase) and an increase in iNOS, leading to the formation of peroxynitrite (Hernández et al., 2011). We have previously reported that obese rats treated with diazoxide displayed reduced levels of lipid peroxidation in both the soleus and EDL muscles. Moreover, it has been observed that the pretreatment with diazoxide contributes to the resistance of muscle tissue against ischemic damage, accompanied by decreased levels of lipid peroxidation (Farahini et al., 2012). Similarly, a decrease in lipid peroxidation levels has been demonstrated in obese rats subjected to exercise (Gómez‐Barroso et al., 2020).

A mechanism to prevent oxidative damage is the increase in endogenous antioxidant enzymes like catalase and glutathione peroxidase, whose synthesis is modified by exercise, diet, and age (Fernández et al., 2009; Miguel‐Dos‐Santos et al., 2021). Parallel to an increment in oxidant levels, the activity of catalase decreased in the hypertensive rats, which was restored with diazoxide and exercise in the EDL and soleus muscles (Figure 7). In healthy individuals, enzymes are expressed in different ways in different organs, depending on the metabolic and functional processes that occur in them. Consistent with our results, reduced activity of antioxidant enzymes has been observed in hypertensive animals (Miguel‐Dos‐Santos et al., 2021). Other studies corroborate these findings, demonstrating that both the activity and gene expression of these enzymes are reduced in a model of renovascular hypertension (Miguel‐Dos‐Santos et al., 2021). Training has been shown to increase the activity of the superoxide dismutase and catalase in the heart and kidney in a model of renovascular hypertension. Resistance training increases antioxidant enzymes in skeletal and cardiac muscle (Maia et al., 2015; Miguel‐Dos‐Santos et al., 2021; Nishi et al., 2010; Roumeliotis et al., 2019; Toklu et al., 2010). Moreover, superoxide dismutase and catalase activity have been shown to decrease muscle tissue during ischemia–reperfusion. Pretreatment with diazoxide in animals increased superoxide dismutase and catalase activity after ischemia–reperfusion injury (Cardoso et al., 2013).

Regarding glutathione, the GSH/GSSG ratio in soleus and EDL muscles was decreased in hypertensive rats (Figure 6). The decrease in GSH content suggests that HTN affects antioxidant capacity. Glutathione levels have been reported to increase in diazoxide‐treated skeletal muscle during ischemia–reperfusion (Moghtadaei et al., 2012). Similarly, exercise may have the same effect during HTN as it improves antioxidant activity (Nyberg et al., 2015). Glutathione plays a vital role in NO metabolism as peroxynitrite reacts with GSH to form S‐nitrosothiols (RSNO), which subsequently release NO over a prolonged period of time, thereby extending the half‐life of NO and avoiding the reaction of NO with superoxide (Robaczewska et al., 2016). The role of glutathione in NO availability reflects that this antioxidant has a role in maintaining endothelial function (Robaczewska et al., 2016), and this is likely important for improving blood flow and oxygen utilization in skeletal muscle contraction (Nyberg et al., 2015).

Previous studies suggested that redox imbalance is associated with the pathogenesis of HTN due to an imbalance between elevated pro‐oxidant production and/or reduced antioxidant capacity. Our results and previous studies (Gómez‐Barroso et al., 2020; Larsen & Matchkov, 2016) suggest that diazoxide and exercise may be beneficial in the treatment of HTN with the advantage of an improvement in the functionality of skeletal muscle and achieving a better redox balance in the muscle.

In conclusion, diazoxide and exercise improve the function of slow and fast‐type muscle fibers in HTN. Diazoxide and exercise in rats with HTN present a greater magnitude of the hypotensive effect, decrease oxidant levels, improve the redox state of glutathione, increase the catalase antioxidant defense, and improve muscle contraction force, which could have a therapeutic implication in favor of these treatments.

## 5 PERSPECTIVES AND SIGNIFICANCE

Although scientific information is limited in the context of skeletal muscle in hypertensive states, there is great interest in the field to investigate and discover the details of the fatigue process and how oxidative stress is involved in this pathological state. This knowledge could be translated into the design of possible pharmacological or functional therapies for treating skeletal muscle recovery in hypertension, as demonstrated in this study with diazoxide and moderate‐intensity exercise. This research on pathophysiological models of skeletal muscle in the effects of exercise and diazoxide can be investigated with methodological approaches that allow evaluation of the participation of other factors that may be involved with oxidative stress and skeletal muscle functionality.

## AUTHOR CONTRIBUTIONS

EBS: Data curation, Formal analysis, Methodology, Validation, Visualization, Writing—original draft. CJNR: Data curation, Methodology. MGB: Data curation, Methodology, Project administration, Supervision. CCR: Resources, Software, Supervision. ARRO: Formal analysis, Investigation, Supervision, Visualization. SMA: Resources, Supervision, Validation. ASM: Funding acquisition, Project administration, Supervision. RMP: Conceptualization, Funding acquisition, Project administration, Supervision, Validation, Writing—original draft, Writing—review and editing.

## FUNDING INFORMATION

UMSNH‐CIC‐2023 to Rocío Montoya‐Pérez.

## CONFLICT OF INTEREST STATEMENT

The authors have no conflict of interest to declare.

## Supporting information


Data S1.

## Data Availability

All data are included in the document.
