# Supplementary material for: Diazoxide and moderate‐intensity exercise improve skeletal muscle function by decreasing oxidants and enhancing antioxidant defenses in hypertensive male rats
Source: Physiol Rep. 2024 Apr 23;12(8):e16026. doi: 10.14814/phy2.16026 (PMC11039411; doi:10.14814/phy2.16026)
Supplement: Supplementary file 1 — Data S1. [file PHY2-12-e16026-s001.docx]

**Supplementary material**

***Original research article***

**Diazoxide and moderate-intensity exercise improve skeletal muscle function by decreasing oxidants and enhancing antioxidant defenses in hypertensive male rats**

**Supplementary tables**

The main effects of each factor (Hypertension x Exercise x Drug) on the dependent variables were examined, as well as interactions between the factors to identify combined effects among two or more factors that influence the dependent variable.

**Supplementary Table S1. Table of univariate results for the effect of NaCl on systolic blood pressure**

|  | Degr. of | SBP | SBP | SBP | SBP |
| --- | --- | --- | --- | --- | --- |
|  | Freedom | SS | MS | F | p |
| Weeks | 4 | 5506 | 1377 | F (4, 16) = 37.30 | 0.0001 |
| HTN | 1 | 7248 | 7248 | F (1, 4) = 174.5 | 0.0002 |
| Weeks*HTN | 4 | 5417 | 1354 | F (4, 16) = 25.87 | 0.0001 |
| Interaction: Weeks x Subject | 16 | 590.5 | 36.91 |  |  |
| Interaction: HTN x Subject | 4 | 166.1 | 41.53 |  |  |
| Subject | 4 | 46.68 | 11.67 |  |  |
| Residual | 16 | 837.5 | 52.34 |  |  |

**Supplementary Table S2. Table of univariate results for the effect of NaCl on systolic blood pressure**

|  | Degre. of | BP | DBP | DBP | DBP |
| --- | --- | --- | --- | --- | --- |
|  | Freedom | SS | MS | F | p |
| Weeks | 4 | 1709 | 427.2 | F (4, 16) = 7.547 | 0.0013 |
| HTN | 1 | 6751 | 6751 | F (1, 4) = 36.83 | 0.0037 |
| Interaction: Weeks*HTN | 4 | 2797 | 699.4 | F (4, 16) = 8.183 | 0.0009 |
| Interaction: Weeks x Subject | 16 | 905.6 | 56.6 |  |  |
| Interaction: HTN x Subject | 4 | 733.3 | 183.3 |  |  |
| Subject | 4 | 218.8 | 54.7 |  |  |
| Residual | 16 | 1368 | 85.47 |  |  |

**Supplementary Table S3. Table of univariate results of systolic blood pressure**

|  | Degr. of | SBP | SBP | SBP | SBP |
| --- | --- | --- | --- | --- | --- |
|  | Freedom | SS | MS | F | p |
| Intercept | 1 | 813105.2 | 813105.2 | 9516.959 | 0.000000 |
| HTN | 1 | 19758.0 | 19758.0 | 231.257 | 0.000000 |
| EX | 1 | 189.2 | 189.2 | 2.215 | 0.146484 |
| DZX | 1 | 5688.2 | 5688.2 | 66.578 | 0.000000 |
| HTN*EX | 1 | 416.0 | 416.0 | 4.869 | 0.034632 |
| HTN*DZX | 1 | 1677.0 | 1677.0 | 19.629 | 0.000103 |
| EX*DZX | 1 | 648.0 | 648.0 | 7.585 | 0.009625 |
| HTN*EX*DZX | 1 | 265.2 | 265.2 | 3.104 | 0.087637 |
| Error | 32 | 2734.0 | 85.4 |  |  |
| Total | 39 | 31375.8 |  |  |  |

**Supplementary Table S4. Table of univariate results of diastolic blood pressure**

|  | Degr. of | DBP | DBP | DBP | DBP |
| --- | --- | --- | --- | --- | --- |
|  | Freedom | SS | MS | F | p |
| Intercept | 1 | 422302.5 | 422302.5 | 3850.929 | 0.000000 |
| HTN | 1 | 17222.5 | 17222.5 | 157.050 | 0.000000 |
| EX | 1 | 122.5 | 122.5 | 1.117 | 0.298466 |
| DZX | 1 | 1960.0 | 1960.0 | 17.873 | 0.000184 |
| HTN*EX | 1 | 372.1 | 372.1 | 3.393 | 0.074752 |
| HTN*DZX | 1 | 6.4 | 6.4 | 0.058 | 0.810647 |
| EX*DZX | 1 | 672.4 | 672.4 | 6.132 | 0.018756 |
| HTN*EX*DZX | 1 | 270.4 | 270.4 | 2.466 | 0.126189 |
| Error | 32 | 3509.2 | 109.7 |  |  |
| Total | 39 | 24135.5 |  |  |  |

**Supplementary Table S5. Table of univariate results of weight**

|  | Degr. of | WEIGHT | WEIGHT | WEIGHT | WEIGHT |
| --- | --- | --- | --- | --- | --- |
|  | Freedom | SS | MS | F | p |
| Intercept | 1 | 6915586 | 6915586 | 13299.20 | 0.000000 |
| HTN | 1 | 42120 | 42120 | 81.00 | 0.000000 |
| EX | 1 | 11357 | 11357 | 21.84 | 0.000051 |
| DZX | 1 | 1464 | 1464 | 2.82 | 0.103095 |
| HTN*EX | 1 | 2310 | 2310 | 4.44 | 0.042967 |
| HTN*DZX | 1 | 144 | 144 | 0.28 | 0.601852 |
| EX*DZX | 1 | 1742 | 1742 | 3.35 | 0.076501 |
| HTN*EX*DZX | 1 | 240 | 240 | 0.46 | 0.501702 |
| Error | 32 | 16640 | 520 |  |  |
| Total | 39 | 76018 |  |  |  |

**Supplementary Table S6. Table of univariate results of glucose**

|  | Degr. of | GLUCOSE | GLUCOSE | GLUCOSE | GLUCOSE |
| --- | --- | --- | --- | --- | --- |
|  | Freedom | SS | MS | F | p |
| Intercept | 1 | 732.9928 | 732.9928 | 4238.208 | 0.000000 |
| HTN | 1 | 0.3080 | 0.3080 | 1.781 | 0.191459 |
| EX | 1 | 3.3466 | 3.3466 | 19.350 | 0.000113 |
| DZX | 1 | 1.6120 | 1.6120 | 9.321 | 0.004535 |
| HTN*EX | 1 | 0.0018 | 0.0018 | 0.011 | 0.918878 |
| HTN*DZX | 1 | 1.1323 | 1.1323 | 6.547 | 0.015438 |
| EX*DZX | 1 | 0.5593 | 0.5593 | 3.234 | 0.081563 |
| HTN*EX*DZX | 1 | 4.0386 | 4.0386 | 23.351 | 0.000032 |
| Error | 32 | 5.5344 | 0.1729 |  |  |
| Total | 39 | 16.5331 |  |  |  |

**Supplementary Table S7. Table of univariate results of KITT**

|  | Degr. of | KITT | KITT | KITT | KITT |
| --- | --- | --- | --- | --- | --- |
|  | Freedom | SS | MS | F | p |
| Intercept | 1 | 298.0123 | 298.0123 | 308.1071 | 0.000000 |
| HTN | 1 | 0.2712 | 0.2712 | 0.2804 | 0.600117 |
| EX | 1 | 2.7996 | 2.7996 | 2.8944 | 0.098585 |
| DZX | 1 | 2.1742 | 2.1742 | 2.2479 | 0.143602 |
| HTN*EX | 1 | 0.1380 | 0.1380 | 0.1427 | 0.708133 |
| HTN*DZX | 1 | 1.1359 | 1.1359 | 1.1744 | 0.286599 |
| EX*DZX | 1 | 3.6888 | 3.6888 | 3.8137 | 0.059627 |
| HTN*EX*DZX | 1 | 0.1528 | 0.1528 | 0.1580 | 0.693618 |
| Error | 32 | 30.9516 | 0.9672 |  |  |
| Total | 39 | 41.3121 |  |  |  |

**Supplementary Table S8. Table of univariate results of total tension EDL**

|  | Degr. of | TOTAL TENSION EDL | TOTAL TENSION EDL | TOTAL TENSION EDL | TOTAL TENSION EDL |
| --- | --- | --- | --- | --- | --- |
|  | Freedom | SS | MS | F | p |
| Intercept | 1 | 11649981 | 11649981 | 1617.654 | 0.000000 |
| HTN | 1 | 893943 | 893943 | 124.128 | 0.000000 |
| EX | 1 | 462029 | 462029 | 64.155 | 0.000000 |
| DZX | 1 | 542730 | 542730 | 75.361 | 0.000000 |
| HTN*EX | 1 | 5361 | 5361 | 0.744 | 0.394665 |
| HTN*DZX | 1 | 27961 | 27961 | 3.883 | 0.057494 |
| EX*DZX | 1 | 147 | 147 | 0.020 | 0.887428 |
| HTN*EX*DZX | 1 | 15643 | 15643 | 2.172 | 0.150309 |
| Error | 32 | 230457 | 7202 |  |  |
| Total | 39 | 2178271 |  |  |  |

**Supplementary Table S9. Table of univariate results of peak tension EDL**

|  | Degr. of | PEAK TENSION EDL | PEAK TENSION EDL | PEAK TENSION EDL | PEAK TENSION EDL |
| --- | --- | --- | --- | --- | --- |
|  | Freedom | SS | MS | F | p |
| Intercept | 1 | 3826219 | 3826219 | 1342.760 | 0.000000 |
| HTN | 1 | 115944 | 115944 | 40.689 | 0.000000 |
| EX | 1 | 214106 | 214106 | 75.138 | 0.000000 |
| DZX | 1 | 319769 | 319769 | 112.219 | 0.000000 |
| HTN*EX | 1 | 1950 | 1950 | 0.684 | 0.414265 |
| HTN*DZX | 1 | 18 | 18 | 0.006 | 0.937721 |
| EX*DZX | 1 | 9921 | 9921 | 3.481 | 0.071249 |
| HTN*EX*DZX | 1 | 13686 | 13686 | 4.803 | 0.035807 |
| Error | 32 | 91185 | 2850 |  |  |
| Total | 39 | 766577 |  |  |  |

**Supplementary Table S10. Table of univariate results of total tension soleus**

|  | Degr. of | TOTAL TENSION SOL | TOTAL TENSION SOL | TOTAL TENSION SOL | TOTAL TENSION SOL |
| --- | --- | --- | --- | --- | --- |
|  | Freedom | SS | MS | F | p |
| Intercept | 1 | 3587836 | 3587836 | 1277.734 | 0.000000 |
| HTN | 1 | 154110 | 154110 | 54.883 | 0.000000 |
| EX | 1 | 103628 | 103628 | 36.905 | 0.000001 |
| DZX | 1 | 140492 | 140492 | 50.033 | 0.000000 |
| HTN*EX | 1 | 14339 | 14339 | 5.106 | 0.030779 |
| HTN*DZX | 1 | 4 | 4 | 0.001 | 0.971493 |
| EX*DZX | 1 | 66 | 66 | 0.023 | 0.879437 |
| HTN*EX*DZX | 1 | 5160 | 5160 | 1.838 | 0.184703 |
| Error | 32 | 89855 | 2808 |  |  |
| Total | 39 | 507653 |  |  |  |

**Supplementary Table S11. Table of univariate results of peak tension soleus**

|  | Degr. of | PEAK TENSION SOL | PEAK TENSION SOL | PEAK TENSION SOL | PEAK TENSION SOL |
| --- | --- | --- | --- | --- | --- |
|  | Freedom | SS | MS | F | p |
| Intercept | 1 | 1283414 | 1283414 | 1480.147 | 0.000000 |
| HTN | 1 | 39423 | 39423 | 45.466 | 0.000000 |
| EX | 1 | 72287 | 72287 | 83.368 | 0.000000 |
| DZX | 1 | 81637 | 81637 | 94.151 | 0.000000 |
| HTN*EX | 1 | 7208 | 7208 | 8.312 | 0.006983 |
| HTN*DZX | 1 | 3041 | 3041 | 3.507 | 0.070272 |
| EX*DZX | 1 | 783 | 783 | 0.903 | 0.349160 |
| HTN*EX*DZX | 1 | 592 | 592 | 0.682 | 0.414951 |
| Error | 32 | 27747 | 867 |  |  |
| Total | 39 | 232717 |  |  |  |

**Supplementary Table S12. Table of univariate results of fatigue time EDL**

|  | Degr. of | FATIGUE EDL | FATIGUE EDL | FATIGUE EDL | FATIGUE EDL |
| --- | --- | --- | --- | --- | --- |
|  | Freedom | SS | MS | F | p |
| Intercept | 1 | 1857179 | 1857179 | 1284.167 | 0.000000 |
| HTN | 1 | 52201 | 52201 | 36.095 | 0.000001 |
| EX | 1 | 97318 | 97318 | 67.292 | 0.000000 |
| DZX | 1 | 144841 | 144841 | 100.152 | 0.000000 |
| HTN*EX | 1 | 391 | 391 | 0.270 | 0.606840 |
| HTN*DZX | 1 | 2356 | 2356 | 1.629 | 0.210995 |
| EX*DZX | 1 | 29757 | 29757 | 20.576 | 0.000076 |
| HTN*EX*DZX | 1 | 189 | 189 | 0.131 | 0.719938 |
| Error | 32 | 46279 | 1446 |  |  |
| Total | 39 | 373332 |  |  |  |

**Supplementary Table S13. Table of univariate results of fatigue time soleus**

|  | Degr. of | FATIGUE SOL | FATIGUE SOL | FATIGUE SOL | FATIGUE SOL |
| --- | --- | --- | --- | --- | --- |
|  | Freedom | SS | MS | F | p |
| Intercept | 1 | 2278630 | 2278630 | 1831.770 | 0.000000 |
| HTN | 1 | 358156 | 358156 | 287.918 | 0.000000 |
| EX | 1 | 69306 | 69306 | 55.714 | 0.000000 |
| DZX | 1 | 73702 | 73702 | 59.249 | 0.000000 |
| HTN*EX | 1 | 7263 | 7263 | 5.839 | 0.021558 |
| HTN*DZX | 1 | 2873 | 2873 | 2.310 | 0.138397 |
| EX*DZX | 1 | 13141 | 13141 | 10.564 | 0.002714 |
| HTN*EX*DZX | 1 | 15484 | 15484 | 12.448 | 0.001291 |
| Error | 32 | 39806 | 1244 |  |  |
| Total | 39 | 579731 |  |  |  |

**Supplementary Table S14. Table of univariate results of oxidants EDL**

|  | Degr. of | OXIDANTS EDL | OXIDANTS EDL | OXIDANTS EDL | OXIDANTS EDL |
| --- | --- | --- | --- | --- | --- |
|  | Freedom | SS | MS | F | p |
| Intercept | 1 | 32473.08 | 32473.08 | 1372.349 | 0.000000 |
| HTN | 1 | 2987.88 | 2987.88 | 126.271 | 0.000000 |
| EX | 1 | 89.68 | 89.68 | 3.790 | 0.060382 |
| DZX | 1 | 944.71 | 944.71 | 39.924 | 0.000000 |
| HTN*EX | 1 | 692.00 | 692.00 | 29.245 | 0.000006 |
| HTN*DZX | 1 | 419.19 | 419.19 | 17.715 | 0.000194 |
| EX*DZX | 1 | 1.27 | 1.27 | 0.054 | 0.818433 |
| HTN*EX*DZX | 1 | 125.69 | 125.69 | 5.312 | 0.027820 |
| Error | 32 | 757.20 | 23.66 |  |  |
| Total | 39 | 6017.62 |  |  |  |

**Supplementary Table S15. Table of univariate results of oxidants soleus**

|  | Degr. of | OXIDANTS SOL | OXIDANTS SOL | OXIDANTS SOL | OXIDANTS SOL |
| --- | --- | --- | --- | --- | --- |
|  | Freedom | SS | MS | F | p |
| Intercept | 1 | 22816.37 | 22816.37 | 1268.400 | 0.000000 |
| HTN | 1 | 2735.26 | 2735.26 | 152.057 | 0.000000 |
| EX | 1 | 30.75 | 30.75 | 1.710 | 0.200339 |
| DZX | 1 | 609.87 | 609.87 | 33.904 | 0.000002 |
| HTN*EX | 1 | 527.93 | 527.93 | 29.348 | 0.000006 |
| HTN*DZX | 1 | 530.56 | 530.56 | 29.495 | 0.000006 |
| EX*DZX | 1 | 16.00 | 16.00 | 0.889 | 0.352735 |
| HTN*EX*DZX | 1 | 4.51 | 4.51 | 0.251 | 0.619821 |
| Error | 32 | 575.63 | 17.99 |  |  |
| Total | 39 | 5030.51 |  |  |  |

**Supplementary Table S16. Table of univariate results of total glutathione EDL**

|  | Degr. of | TOTAL GLUT EDL | TOTAL GLUT EDL | TOTAL GLUT EDL | TOTAL GLUT EDL |
| --- | --- | --- | --- | --- | --- |
|  | Freedom | SS | MS | F | p |
| Intercept | 1 | 397478.9 | 397478.9 | 2384.160 | 0.000000 |
| HTN | 1 | 3338.1 | 3338.1 | 20.022 | 0.000091 |
| EX | 1 | 3900.8 | 3900.8 | 23.398 | 0.000032 |
| DZX | 1 | 1910.5 | 1910.5 | 11.460 | 0.001896 |
| HTN*EX | 1 | 166.1 | 166.1 | 0.996 | 0.325691 |
| HTN*DZX | 1 | 708.5 | 708.5 | 4.250 | 0.047464 |
| EX*DZX | 1 | 206.1 | 206.1 | 1.236 | 0.274491 |
| HTN*EX*DZX | 1 | 497.4 | 497.4 | 2.983 | 0.093774 |
| Error | 32 | 5334.9 | 166.7 |  |  |
| Total | 39 | 16062.3 |  |  |  |

**Supplementary Table S17. Table of univariate results of GSSG EDL**

|  | Degr. of | GSSG EDL | GSSG EDL | GSSG EDL | GSSG EDL |
| --- | --- | --- | --- | --- | --- |
|  | Freedom | SS | MS | F | p |
| Intercept | 1 | 492176.2 | 492176.2 | 4719.257 | 0.000000 |
| HTN | 1 | 966.2 | 966.2 | 9.264 | 0.004644 |
| EX | 1 | 6082.9 | 6082.9 | 58.326 | 0.000000 |
| DZX | 1 | 5241.1 | 5241.1 | 50.254 | 0.000000 |
| HTN*EX | 1 | 17519.8 | 17519.8 | 167.989 | 0.000000 |
| HTN*DZX | 1 | 5754.9 | 5754.9 | 55.181 | 0.000000 |
| EX*DZX | 1 | 25.0 | 25.0 | 0.240 | 0.627855 |
| HTN*EX*DZX | 1 | 616.0 | 616.0 | 5.907 | 0.020866 |
| Error | 32 | 3337.3 | 104.3 |  |  |
| Total | 39 | 39543.2 |  |  |  |

**Supplementary Table S18. Table of univariate results of GSH EDL**

|  | Degr. of | GSH EDL | GSH EDL | GSH EDL | GSH EDL |
| --- | --- | --- | --- | --- | --- |
|  | Freedom | SS | MS | F | p |
| Intercept | 1 | 359592.1 | 359592.1 | 2615.131 | 0.000000 |
| HTN | 1 | 10100.1 | 10100.1 | 73.453 | 0.000000 |
| EX | 1 | 25783.2 | 25783.2 | 187.508 | 0.000000 |
| DZX | 1 | 17276.8 | 17276.8 | 125.645 | 0.000000 |
| HTN*EX | 1 | 4514.6 | 4514.6 | 32.833 | 0.000002 |
| HTN*DZX | 1 | 9600.7 | 9600.7 | 69.821 | 0.000000 |
| EX*DZX | 1 | 436.3 | 436.3 | 3.173 | 0.084358 |
| HTN*EX*DZX | 1 | 96.4 | 96.4 | 0.701 | 0.408575 |
| Error | 32 | 4400.1 | 137.5 |  |  |
| Total | 39 | 72208.2 |  |  |  |

**Supplementary Table S19. Table of univariate results of ratio EDL**

|  | Degr. of | RATIO EDL | RATIO EDL | RATIO EDL | RATIO EDL |
| --- | --- | --- | --- | --- | --- |
|  | Freedom | SS | MS | F | p |
| Intercept | 1 | 43.33885 | 43.33885 | 617.9773 | 0.000000 |
| HTN | 1 | 0.00239 | 0.00239 | 0.0340 | 0.854838 |
| EX | 1 | 5.89707 | 5.89707 | 84.0875 | 0.000000 |
| DZX | 1 | 4.19920 | 4.19920 | 59.8772 | 0.000000 |
| HTN*EX | 1 | 4.76453 | 4.76453 | 67.9383 | 0.000000 |
| HTN*DZX | 1 | 3.78308 | 3.78308 | 53.9436 | 0.000000 |
| EX*DZX | 1 | 1.55016 | 1.55016 | 22.1041 | 0.000047 |
| HTN*EX*DZX | 1 | 1.93051 | 1.93051 | 27.5276 | 0.000010 |
| Error | 32 | 2.24417 | 0.07013 |  |  |
| Total | 39 | 24.37110 |  |  |  |

**Supplementary Table S20. Table of univariate results of total glutathione soleus**

|  | Degr. of | TOTAL GLUT SOL | TOTAL GLUT SOL | TOTAL GLUT SOL | TOTAL GLUT SOL |
| --- | --- | --- | --- | --- | --- |
|  | Freedom | SS | MS | F | p |
| Intercept | 1 | 438852.6 | 438852.6 | 3206.139 | 0.000000 |
| HTN | 1 | 2764.0 | 2764.0 | 20.193 | 0.000086 |
| EX | 1 | 954.3 | 954.3 | 6.972 | 0.012695 |
| DZX | 1 | 1220.5 | 1220.5 | 8.917 | 0.005382 |
| HTN*EX | 1 | 26.1 | 26.1 | 0.191 | 0.664999 |
| HTN*DZX | 1 | 959.4 | 959.4 | 7.009 | 0.012479 |
| EX*DZX | 1 | 11.3 | 11.3 | 0.082 | 0.776010 |
| HTN*EX*DZX | 1 | 133.0 | 133.0 | 0.972 | 0.331592 |
| Error | 32 | 4380.1 | 136.9 |  |  |
| Total | 39 | 10448.9 |  |  |  |

**Supplementary Table S21. Table of univariate results of GSSG soleus**

|  | Degr. of | GSSG SOL | GSSG SOL | GSSG SOL | GSSG SOL |
| --- | --- | --- | --- | --- | --- |
|  | Freedom | SS | MS | F | p |
| Intercept | 1 | 313886.6 | 313886.6 | 2208.288 | 0.000000 |
| HTN | 1 | 2694.7 | 2694.7 | 18.958 | 0.000128 |
| EX | 1 | 1955.0 | 1955.0 | 13.754 | 0.000788 |
| DZX | 1 | 10349.3 | 10349.3 | 72.811 | 0.000000 |
| HTN*EX | 1 | 1915.3 | 1915.3 | 13.475 | 0.000874 |
| HTN*DZX | 1 | 1234.1 | 1234.1 | 8.682 | 0.005951 |
| EX*DZX | 1 | 133.1 | 133.1 | 0.936 | 0.340524 |
| HTN*EX*DZX | 1 | 5033.6 | 5033.6 | 35.413 | 0.000001 |
| Error | 32 | 4548.5 | 142.1 |  |  |
| Total | 39 | 27863.6 |  |  |  |

**Supplementary Table S22. Table of univariate results of GSH soleus**

|  | Degr. of | GSH SOL | GSH SOL | GSH SOL | GSH SOL |
| --- | --- | --- | --- | --- | --- |
|  | Freedom | SS | MS | F | p |
| Intercept | 1 | 749340.2 | 749340.2 | 1447.547 | 0.000000 |
| HTN | 1 | 29747.5 | 29747.5 | 57.465 | 0.000000 |
| EX | 1 | 39605.2 | 39605.2 | 76.508 | 0.000000 |
| DZX | 1 | 46225.9 | 46225.9 | 89.297 | 0.000000 |
| HTN*EX | 1 | 211.2 | 211.2 | 0.408 | 0.527539 |
| HTN*DZX | 1 | 6605.1 | 6605.1 | 12.759 | 0.001145 |
| EX*DZX | 1 | 2274.5 | 2274.5 | 4.394 | 0.044066 |
| HTN*EX*DZX | 1 | 3.3 | 3.3 | 0.006 | 0.936849 |
| Error | 32 | 16565.2 | 517.7 |  |  |
| Total | 39 | 141237.8 |  |  |  |

**Supplementary Table S23. Table of univariate results of ratio soleus**

|  | Degr. of | RATIO SOL | RATIO SOL | RATIO SOL | RATIO SOL |
| --- | --- | --- | --- | --- | --- |
|  | Freedom | SS | MS | F | p |
| Intercept | 1 | 134.8507 | 134.8507 | 570.9381 | 0.000000 |
| HTN | 1 | 0.0170 | 0.0170 | 0.0721 | 0.790007 |
| EX | 1 | 6.8157 | 6.8157 | 28.8568 | 0.000007 |
| DZX | 1 | 19.1956 | 19.1956 | 81.2712 | 0.000000 |
| HTN*EX | 1 | 0.2377 | 0.2377 | 1.0065 | 0.323270 |
| HTN*DZX | 1 | 2.6892 | 2.6892 | 11.3855 | 0.001953 |
| EX*DZX | 1 | 0.6546 | 0.6546 | 2.7716 | 0.105711 |
| HTN*EX*DZX | 1 | 0.4279 | 0.4279 | 1.8116 | 0.187779 |
| Error | 32 | 7.5581 | 0.2362 |  |  |
| Total | 39 | 37.5958 |  |  |  |

**Supplementary Table S24. Table of univariate results of catalase EDL**

|  | Degr. of | CAT EDL | CAT EDL | CAT EDL | CAT EDL |
| --- | --- | --- | --- | --- | --- |
|  | Freedom | SS | MS | F | p |
| Intercept | 1 | 121328.7 | 121328.7 | 953.0886 | 0.000000 |
| HTN | 1 | 3660.5 | 3660.5 | 28.7544 | 0.000007 |
| EX | 1 | 10848.1 | 10848.1 | 85.2167 | 0.000000 |
| DZX | 1 | 6506.5 | 6506.5 | 51.1115 | 0.000000 |
| HTN*EX | 1 | 24.5 | 24.5 | 0.1923 | 0.663954 |
| HTN*DZX | 1 | 6269.9 | 6269.9 | 49.2530 | 0.000000 |
| EX*DZX | 1 | 148.3 | 148.3 | 1.1650 | 0.288499 |
| HTN*EX*DZX | 1 | 1989.2 | 1989.2 | 15.6258 | 0.000400 |
| Error | 32 | 4073.6 | 127.3 |  |  |
| Total | 39 | 33520.6 |  |  |  |

**Supplementary Table S25. Table of univariate results of catalase soleus**

|  | Degr. of | CAT SOL | CAT SOL | CAT SOL | CAT SOL |
| --- | --- | --- | --- | --- | --- |
|  | Freedom | SS | MS | F | p |
| Intercept | 1 | 49196.45 | 49196.45 | 763.7302 | 0.000000 |
| HTN | 1 | 1561.15 | 1561.15 | 24.2354 | 0.000025 |
| EX | 1 | 4529.58 | 4529.58 | 70.3176 | 0.000000 |
| DZX | 1 | 6429.10 | 6429.10 | 99.8059 | 0.000000 |
| HTN*EX | 1 | 146.06 | 146.06 | 2.2675 | 0.141921 |
| HTN*DZX | 1 | 494.87 | 494.87 | 7.6824 | 0.009215 |
| EX*DZX | 1 | 447.02 | 447.02 | 6.9395 | 0.012884 |
| HTN*EX*DZX | 1 | 26.36 | 26.36 | 0.4092 | 0.526909 |
| Error | 32 | 2061.31 | 64.42 |  |  |
| Total | 39 | 15695.45 |  |  |  |
